# Assessment of Inflammatory Markers in Children with Cow’s Milk Allergy Treated with a Milk-Free Diet

**DOI:** 10.3390/nu13041057

**Published:** 2021-03-24

**Authors:** Jadwiga Ambroszkiewicz, Joanna Gajewska, Magdalena Chełchowska, Grażyna Rowicka

**Affiliations:** 1 Department of Screening Tests and Metabolic Diagnostics, Institute of Mother and Child, Kasprzaka 17A, 01-211 Warsaw, Poland; joanna.gajewska@imid.med.pl (J.G.); magdalena.chelchowska@imid.med.pl (M.C.); 2 Department of Nutrition, Institute of Mother and Child, Kasprzaka 17A, 01-211 Warsaw, Poland; grazyna.rowicka@imid.med.pl

**Keywords:** inflammation, cytokines, calprotectin, pro-inflammatory adipokines, cow’s milk allergy

## Abstract

Background: The aim of the study was to establish whether the use of a strict milk-free diet in children with cow’s milk allergy, resulting in the resolution of clinical symptoms of the disease, also extinguishes the inflammatory reaction induced by the allergy. Methods: We examined 64 children (aged 3–6 years) with a diagnosed cow’s milk allergy who had been treated with an elimination diet for at least six months and showed remission of the disease’s clinical symptoms as a result of the treatment. The control group consisted of 30 healthy children of the same age following an unrestricted age-appropriate diet. Concentrations of cytokines, calprotectin, and adipokines (leptin, resistin, chemerin, neutrophilic lipocalin associated with gelatinase—NGAL) were determined in the serum samples obtained from the studied children by immunoenzymatic assays. Results: Patients with CMA had significantly higher median values of serum IL-6, TNF-α, resistin, chemerin and NGAL in comparison to the healthy children (*p* < 0.05, *p* < 0.001, *p* < 0.05, *p* < 0.01, *p* < 0.001, respectively). Serum concentrations of IL-10, leptin, calprotectin and CRP as well as in WBC count were in the same range in both studied groups. We observed direct statistically significant correlations between levels of IL-10 and CRP (*p* = 0.005), IL-10 and WBC (*p* = 0.045), TNF-α and WBC (*p* = 0.038), calprotectin and WBC (*p* < 0.001), chemerin and CRP (*p* < 0.001) as well as between NGAL and WBC (*p* = 0.002) in children with CMA. Conclusion: The use of a strict milk-free diet by children with CMA, resulting in the resolution of clinical symptoms of the disease, does not seem to extinguish the inflammation induced by the allergy. The findings of this study—elevated IL-6, TNF-α, resistin, chemerin and NGAL levels in patients with CMA—suggest that these parameters seem to be involved in the generation of a low-grade proinflammatory environment observed in cow‘s milk allergy and could be used to monitor the effectiveness of treatment.

## 1. Introduction

Cow’s milk allergy (CMA) is one of the most common food allergy forms in children, affecting about 2–5% of infants and young children [1,2]. CMA is caused by an immune reaction to one or more milk proteins and can be immunoglobulin (Ig)E mediated, non-IgE mediated or mixed type [3,4]. The only effective treatment for CMA is eliminating cows’ milk from the diet and replacing it with appropriate substitutes, including casein or whey extensively hydrolyzed formulas, amino-acid-based formulas and for some children soy products [5,6]. Most children with CMA develop tolerance to cow’s milk proteins but in some cases the symptoms of allergy persist for many years. Currently determined parameters are of limited value in this disease treatment as they do not fully reflect inflammatory processes. Therefore, searching for new potential biomarkers allowing both detection and monitoring the dynamics of the inflammatory process is necessary [7].

Disturbances in the regulation of the immune response of the Th1/Th2 cells to antigen play a key role in the patophysiology of allergic diseases including food allergy. Lately, special attention has been paid to cytokines as the main regulators of cow’s milk allergy. Among them, IL-6, IL-10 and TNF-α play essential role. TNF-α with its pro-inflammatory properties can promote Th1 response, whereas IL-10 plays a role in Th1/Th2 cross-regulation. IL-10 produced by Th2 cells directly suppresses Th1 cells and can indirectly suppress Th2 cells activity [8,9,10].

The link between inflammation and adipose tissue has not yet been understood [11,12]. In recent years, attention has been paid to adipokines as one of the mediators responsible for the low grade—inflammation involved in the pathomechanism of several diseases [13,14,15]. Adipokines acting at different molecular levels may be the regulators of multiple responses linking inflammation, immunity, and metabolism. Among them, well-known adipokines such as leptin and resistin are of great interest. They participate in the inflammatory response leading to an increased expression of several pro-inflammatory cytokines (IL-1, IL-6, IL-12, TNF-α) from macrophages and their levels increase especially during chronic inflammation [16,17,18,19,20,21]. Chemerin is another protein classified both as an adipokine (because of its role in adipocyte differentiation) and as a chemoattractant. It acts through a G protein-coupled receptor [22,23,24]. Due to the chemotactic properties related to specialized leukocyte populations, chemerin is considered an influential marker of low intensity chronic inflammation and macrophages accumulation [25,26,27].

Additionally, neutrophilic lipocalin associated with gelatinase (NGAL) which from a metabolic standpoint is also considered to be an adipokine (due to its high expression in adipose tissue) plays a possible role in neutrophilic inflammation [28,29]. Binding to matrix metalloproteinase (MMP)-9 released from neutrophils it can also exhibit activity associated with its siderophore- and iron-binding properties [30]. It is known that Toll-like receptor (TLR) activation is involved in the regulation of NGAL activity [31], however, the exact mechanism of NGAL involvement in the inflammatory response is still unclear. The utility of NGAL has been tested in various clinical situations; its increased levels are reported in patients with kidney and rheumatic diseases, pancreatitis, chronic obstructive pulmonary disease, sepsis, and malignancy [32,33].

Calprotectin, reported as an early signal mediating the cytokine cascade, is involved in an organism’s first response to inflammation. It acts through TLR-4 and activates nuclear factor-κB and other transcription factors, leading to increased production of metalloproteinases and proinflammatory cytokines [34,35,36]. Patients who suffer from diseases characterized by chronic inflammation had elevated plasma calprotectin [37].

Thus far, there has been limited research on serum pro-inflammatory cytokine and adipokine concentrations as well as calprotectin levels—essential modulators of inflammatory reaction and immunity—in children with cow’s milk allergy. The aim of the study was to investigate whether the use of a strict milk-free diet in children with cow‘s milk allergy, resulting in the resolution of clinical symptoms of the disease, also extinguishes the inflammatory reaction induced by the allergy. For that purpose, we examined serum concentrations of cytokines and adipokines related to inflammatory processes and their correlations with routinely determined parameters such as: white blood cell (WBC) count and C-reactive protein (CRP).

## 2. Materials and Methods

### 2.1. Subjects

This study was approved by the Ethics Committee of the Institute of Mother and Child (Prot. No. 19/2018) and was in accordance with the Helsinki Declaration of Principles. Written informed consent was obtained from all children’s parents. Children were recruited between May 2018 and July 2019 from a group of consecutive patients attending the Institute of Mother and Child in Warsaw (Poland). We examined prepubertal children with cow’s milk allergy treated with a dairy-free diet (n = 64) who remained under medical and dietary care. Cow’s milk protein allergy was diagnosed based on family history of allergy, clinical symptoms suggestive of allergy, measurement of serum cow’s milk-specific IgE concentrations (CM-specific IgE levels of less than 0.35 IU/mL were classified as undetectable), resolution of clinical symptoms after a 4-week diagnostic dairy-free diet. In children with IgE-mediated allergy, the therapeutic diet was continued. While in children with non-IgE-mediated CMA, allergy to cow’s milk was confirmed by re-introduction of dairy products to the diet which resulted in clinical symptoms.

The inclusion criteria were: (a) diagnosed CMA, (b) following a milk-free diet for at least six months resulting in the disappearance of clinical symptoms of allergy, (c) being in the prepubertal period (determined according to the Tanner scale). Patients with acute and chronic infections were excluded. None of the children enrolled in the study were receiving an antibiotic/probiotic at the time of eligibility, time since the last infection treated both symptomatically or requiring antibiotic therapy was at least 3 months. The subjects with CMA had clinical symptoms from one system: gastrointestinal (12/64), skin (7/64), respiratory (30/64) or from many systems (15/64). Children were treated with casein or whey extensively hydrolyzed formulas (54%), soy-based formulas (26%) and plant-based milk substitutes (20%). We divided CMA patients into the IgE-dependent and IgE-independent subgroups resulting from the classification of immune reactions underlying food allergy. There were 41 (64%) IgE-mediated CMA and 23 (36%) non-IgE-mediated CMA patients. In our study, the levels of biochemical inflammatory markers were assessed in children with CMA who had remained on a strict elimination diet for at least 6 months, and 6 to 12 months had passed since the last food provocation attempt.

The control group included prepubertal healthy children (n = 30) within the same age range as the CMA group—with a negative family history of allergy, no clinical symptoms suggestive of allergy, no abnormalities on physical examination, and following a traditional age-appropriate unrestricted diet. These were children who came to the gastroenterology out-patient clinic for evaluation of dietary balance.

All studied children were followed up as outpatients, were normal weight children, and were not taking anti-inflammatory, anti-allergic or immune-modulating drugs/supplements (except for vitamin D supplementation) for at least three months to avoid their influence on the concentrations of biochemical markers of inflammation. All children with CMA were supplemented with vitamin D at a dose of 1000 (500–2000) IU/day depending on body weight and vitamin D supply in the diet. Healthy children (except for 5 cases) were also supplemented with vitamin D at a dose of 1000 (500–1500) IU/day.

### 2.2. Methods

Both allergic and healthy children had physical examinations performed, including height and weight measurements. Body mass index (BMI) was calculated as body weight (kg) divided by height squared (m^2^). The obtained BMI value was related to age-and-gender specific BMI centile charts and BMI z-scores were calculated [38].

For biochemical analyses, 3 mL of venous blood was taken in the morning hours. White blood cell count was determined as part of a routine test. In the serum obtained after centrifugation (1000× *g*, 10 min), the CRP concentration was determined by standard laboratory methods using the biochemical Cobas Integra analyzer (Roche Diagnostics, Switzerland). The rest of the serum was frozen (−20 °C) in portions and stored (no longer than 2 months) for later examination of cytokines and adipokines using enzyme-linked immunosorbent assay (ELISA) kits according to the manufacturer’s instructions. All samples were tested in duplicate.

The concentration of IL-6 was assessed using High Sensitivity IL-6 (hs IL-6) Human ELISA kits from Abcam (Cambridge, UK). The lowest detection limit was 0.81 pg/mL, intra-assay and inter-assay coefficient of variations (CV) were 4.4%, and 9.1%, respectively. Serum IL-10 level was detected using High Sensitivity IL-10 Human ELISA kits from Bender MedSystems GmbH (Vienna, Austria). The minimal detection limit of this assay was 0.05 pg/mL, intra-assay and inter-assay CVs were 6.8% and 7.5%, respectively. The concentration of TNF-α was assessed using kits from DRG Instruments GmbH (Marburg, Germany) with the limit of detection of 0.7 pg/mL, intra-assay precision between 4.6–6.6%, and inter-assay CV: 3.3–4.5%. The concentration of serum calprotectin was measured using kits from Calpro AS (Lysaker, Norway). This assay’s minimal detection limit was 5 ng/mL, intra-assay and inter-assay precision CVs were between 3.6-5.0% and 4.8 = 7.9%, respectively.

Leptin concentration was determined using kits from DRG Instruments GmbH (Marburg, Germany) with the lower detection limit of 0.7 ng/mL, intra-assay CV ranging between 4.2–7.3% and inter-assay CV between 3.7–9.1%. Circulating resistin was measured using kits from AdipoGen Life Science (Liestal, Switzerland) with the limit of detection of 0.1 ng/mL, intra-assay CV between 2.9 and 5.2%, and inter-assay CV between 4.2–7.2%. Chemerin concentration was assessed using a kit from Mediagnost (Reutlingen, Germany), the intra-assay and inter-assay CVs were 2.0–2.8% and 4.4–5.9%, respectively. The analytical sensitivity of this assay is 5 pg/mL. NGAL level was determined using a kit from BioPorto Diagnostics A/S (Hellerup, Denmark), with within assay variability less than 10% and between assay variability less than 12%. This assay‘s minimal detection limit was 1.4 pg/mL.

### 2.3. Statistical Analysis

Statistical analyses were performed using IBM-SPSS software version 23.0 (SPSS INC., Chicago, IL, USA). The Kolmogorov–Smirnov test was used to test the normality of variable distribution. Baseline characteristics were presented as mean±SD for the variables with normal distribution or as median values and interquartile range (IQR) for non-normally distributed variables. Student‘s t-test for normally distributed variables and Mann–Whitney U test for non-parametric variables were applied to evaluate differences between the groups regarding anthropometric and biochemical parameters. Univariate correlation analysis was performed using the Spearman test. A *p* value equal to or less than 0.05 was considered to be statistically significant.

## 3. Results

All the children examined for the purpose of this study were Caucasian. There were 64 children (32.8% girls) aged 3 to 6 years with cow‘s milk allergy treated with a dairy-free diet for at least six months. Their results were compared with group of 30 healthy non-allergic children (33.3% girls) of the same age range, following a traditional unrestricted age-appropriate diet. Although the studied groups of children did not significantly differ in terms of age, the control children were slightly older than the children with CMA. Therefore, they had higher body weight and height. However, the BMI and BMI z-score indexes were comparable in both studied groups (Table 1).

We found statistically significant higher concentrations of IL-6 (*p* = 0.049), TNF-α (*p* < 0.001), resistin (*p* = 0.037), chemerin (*p* = 0.005), and NGAL (*p* < 0.001) in patients with CMA compared with the healthy children (Table 2). We did not observe significant differences in IL-10, calprotectin and leptin concentrations nor in CRP levels and WBC count between the studied groups.

There were no statistically significant differences in levels of biochemical parameters associated with inflammation between the subgroups of children with IgE-mediated CMA and with non-IgE-mediated CMA, except for the concentration of IL-6, which was higher in patients with IgE-mediated CMA (*p* = 0.021) (Table 3). Additionally, we found above two-fold higher eosinophils count in CMA patients compared with healthy children (*p* < 0.001) and significantly higher eosinophils count in IgE mediated subgroup than in non-IgE mediated CMA patients (*p* < 0.001).

We performed an analysis of studied biomarkers in subgroups of patients with different clinical presentations (Table 4). The highest level of IL-10 (about 15%) was noticed in subgroups with gastrointestinal symptoms compared to those with cutaneous or respiratory symptoms (*p* < 0.05). Serum calprotectin was lower in patients with respiratory symptoms than in those with cutaneous or gastrointestinal symptoms by about 20–25% (*p* < 0.05). The values of IL-6 and TNF-α were comparable across the subgroups of patients with CMA. Regarding adipokines, we observed their elevated serum levels in patients with CMA who had clinical symptoms from many systems compared to those with clinical symptoms from one system. Due to the small sample size in the subgroups, these differences did not reach statistical significance, except for NGAL concentrations (*p* = 0.048).

We also analyzed serum concentrations of cytokines, calprotectin and adipokines in patients with different diet durations. We did not observe significant differences in these biomarkers between the three subgroups (Table 5). However, we found a trend towards higher serum IL-6 and lower IL-10 concentrations correlated with longer diet duration. Regarding adipokines, we noticed increased resistin and leptin levels together with longer diet duration (*p* = 0.125, *p* = 0.051, respectively). Due to the small number of patients in the subgroups, these differences did not reach statistical significance (*p* = 095, *p* = 0.089, *p* = 0.125, *p* = 0.051, respectively).

In the group of children with cow’s milk allergy, we found significant positive correlation (*p* < 0.05) between WBC and IL-10, TNF-α and calprotectin levels as well as between the concentration of CRP and IL-10 (Table 6).

We did not observe such relationships in the group of healthy children. In children with CMA, we found a significant direct correlation between chemerin and CRP concentrations (*p* < 0.001) and between NGAL and WBC count (*p* < 0.01) (Table 7).

In the control group, serum resistin was directly correlated with WBC (*p* < 0.01) and with CRP (*p* < 0.01), while chemerin level with WBC (*p* < 0.05) and with CRP (*p* < 0.01). However, leptin concentration was inversely correlated with WBC (*p* < 0.05). Moreover, we observed a significant positive correlation between serum levels of TNF-α and IL-10 (r = 0.290, *p* = 0.022), TNF-α, and chemerin (r = 0.356, *p* = 0.004) and between calprotectin and NGAL (r = 0.432 *p* = 0.001) in patients with CMA. In the group of healthy children we noticed significant relations between the concentration of chemerin and resistin (r = 0.414, *p* = 0.023) and between TNF-α and NGAL (r = 0.513, *p* = 0.004) (data not shown).

Serum levels of 25-hydroxyvitamin D in children from both groups indicated optimal status of this vitamin (31.8 ± 8.4 ng/mL in CMA patients and 31.2 ± 8.0 ng/mL in healthy children).

## 4. Discussion

Considering that cow’s milk allergy is associated with persistent inflammation and the fact that, in some subjects, the symptoms of this disease may last many years, it seems that the search for new markers that can be useful in monitoring the dynamics of the inflammatory process is necessary. The possibility to use cytokines responsible for the allergic process related to CMA has been proposed in previous studies [9]. In the current study we found higher concentrations of IL-6 and TNF-α in patients with CMA compared to healthy children. Moreover, we observed direct correlations between the levels of IL-10 and routinely determined inflammatory markers (CRP and WBC) and between TNF-α and WBC count in CMA children. We did not observe differences in the concentration of inflammatory markers (except for a higher IL-6 level in patients with IgE-mediated CMA) in the groups of children with dependent and independent IgE allergy. Therefore, the type of pathomechanism does not seem to influence the concentrations of the assessed indicators.

In patients with persistent CMA, the exposure to cow’s milk proteins could result in chronic allergic inflammation with its clinical consequences. Mast cells have essential immunoregulatory functions and play a significant role in immediate allergic and inflammatory reactions. Through their production of inflammatory mediators and cytokines, they contribute to the recruitment of eosinophils at the site of inflammation. Mediators released upon mast-cell degranulation, particularly histamine, could stimulate endothelial or epithelial cells to release a potent eosinophil chemoattractant, such as eotaxin. This causes the infiltration of eosinophils, along with basophils, into inflamed tissues. It is known that eosinophils as part of the immune system, when working correctly, could help fight disease and infection. Nevertheless, having too many activated eosinophils may contribute to disease pathology and the self-perpetuating cycle of inflammation. Such dysfunction leads to dysregulation of biological mechanisms involved with eosinophil recruitment and activation [39]. The early phase of CMA clinical manifestations in the IgE-mediated CMA patients is due to the activation of mast cells and basophils to release biologically active substances, such as histamine, interleukins, serine proteases, and TNF-*α*. In our studied patients with CMA, we observed above two-fold higher eosinophils count in the subgroups of IgE-mediated allergic patients than the non-IgE-mediated patients. There was no significant difference regarding eosinophils count between the groups of non IgE-mediated CMA patients and the healthy children. This is not surprising since eosinophilia is one of the markers that can, among others, indicate atopy.

The effects of vitamin D on calcium-phosphate metabolism are relatively well understood. Since vitamin D receptors are present in cells of the immune system, e.g., neutrophils, macrophages, activated B and T lymphocytes and dendritic cells, attention is drawn in the immune response regulation. It has been proven that vitamin D decreases the concentration of proinflammatory cytokines (IL-1, TNFα), simultaneously increasing the concentration of anti-inflammatory cytokines (IL-4, IL-10, IL-5). Thus, an adequate supply of this vitamin to children with inflammatory diseases, including cow’s milk protein allergy, appears be of importance. All children with CMA were supplemented with vitamin D and had a sufficient vitamin D status (serum 25-hydroxyvitamin D above 30 ng/mL). There were no differences in the levels of 25 OH-D in the subgroups of IgE mediated and non IgE mediated CMA patients.

While analyzing the subgroups of patients with different clinical presentations, we observed elevated serum adipokine levels in patients with CMA who had clinical symptoms from many systems compared to those with clinical symptoms from one system. Additionally, we noticed the highest level of IL-10 in patients with gastrointestinal symptoms and decreased serum calprotectin in children with respiratory symptoms. As far as the duration of a milk-free diet is concerned, we observed a trend towards higher serum IL-6 and lower IL-10 concentrations with longer diet duration.

A dysregulated adipokine balance might also be an important link between inflammation and several disorders [13]. Thus, we investigated the levels of pro-inflammatory adipokines (leptin, resistin, chemerin and NGAL), which play a crucial role not only in energy homeostasis but also in the inflammatory and immune reaction. Leptin and resistin being crucial for neutrophil and monocyte/macrophage function, display pro-inflammatory activity. These adipokines stimulate the secretion of IL-6 and TNF-α and influence Th1 and Th2 activity [40]. Previous studies investigating serum levels of pro-inflammatory adipokines in patients with cow‘s milk allergy are limited. Only two of them reported leptin and resistin levels in children with CMA [41,42]. Salmivesi et al. [41] observed that serum leptin and resistin increased significantly while serum IgE decreased during six-month oral immunotherapy for CMA in school-aged children. In turn, Dong et al. [42] reported that children (aged two) with CMA who followed a milk-free diet since the first few months of life had lower (*p* < 0.05) serum leptin compared to healthy children. The results from our study suggest that serum resistin increased but leptin seemed to be unaffected in children with CMA treated with a milk free diet.

Another adipokine, chemerin, was classified as both an adipokine and a chemoattractant for immune cells. Chemerin binding to a receptor (expressed on various immune cells) may regulate chemotaxis towards the site of inflammation. Recently, there have been intense studies on the axis between chemerin and its receptor (chemR23) and its multiple roles in the regulation of cell proliferation, migration and invasion [25,43]. In vitro studies confirmed that chemerin promotes inflammation by recruiting macrophages with pro-inflammatory properties (M1 phenotype) and prevents macrophages from polarizing into an anti-inflammatory phenotype [44,45].

Elevated serum chemerin concentrations and a significant positive correlation of chemerin with the levels of inflammatory markers (CRP, IL-6, TNF-α) were observed in several diseases [25,46]. Chemerin plays an essential role in chronic obstructive pulmonary disease (COPD), where it recruits inflammatory cells to sites of inflammation, leading to endothelial barrier dysfunction, vascular remodeling, and angiogenesis [47]. Patients with severe persistent asthma often had elevated chemerin levels [48]. A high percentage of children with CMA presented respiratory symptoms. Our research showed significantly higher serum concentrations of chemerin in patients with CMA than in healthy children and a significant positive correlation of chemerin concentrations with CRP levels.

Neutrophil gelatinase-associated lipocalin is induced explicitly in damaged nephrons. Therefore, it was initially used for the early detection of acute kidney injury [49]. Recently, NGAL has become an attractive candidate as a marker for various diseases associated with inflammation [28,50]. For the first time, we reported elevated serum concentrations of NGAL in patients with CMA and direct correlations of NGAL levels with chemerin as well as with white blood cell count. There are several reports regarding the association of NGAL with lung diseases [51,52,53,54,55]. In childhood asthma, researchers observed comparable NGAL levels in asthmatic and healthy children [51,52]. However, it is still unknown how NGAL affects this disease. It is not excluded that NGAL may inhibit bacterial growth by binding to siderophores secreted by bacteria [53]. NGAL may also inhibit the inactivation of MMP-9, secreted by activated neutrophils [31].

Some studies mention NGAL as a useful parameter in distinguishing asthma-chronic obstructive (ACO) pulmonary disease from COPD [31,54]. Plasma NGAL levels were lower in the overlapped patients than in patients with COPD. In terms of the pathophysiological mechanism, inflammation in ACO appears to be more complex and may be caused by both neutrophils and eosinophils in contrast to the dominant neutrophilic inflammation in COPD [55]. Recently, researchers reported the association of NGAL with low-grade and high-grade inflammatory conditions [30,56]. Choi et al. [30] assessed 184 adult patients with systemic inflammation who had no renal dysfunction and observed a significant association between NGAL and inflammatory parameter (CRP) in high-grade inflammation, but not in low-grade inflammation states. They suggested that NGAL is a potential indicator for inflammation; however, the role of NGAL according to the intensity of inflammation should be confirmed in future research.

Several previous studies determined serum or fecal calprotectin levels in cases of several inflammatory diseases [57,58,59]. It has been suggested that serum calprotectin (derived predominantly from circulating leukocytes) may be more representative of systemic inflammation, while fecal calprotectin, which is predominantly derived from neutrophils and monocytes/macrophages, may rather reflect intestinal inflammation [60]. Calprotectin, is a protein that is often elevated in the presence of intestinal mucosal inflammation. It was assessed as a potential marker of inflammation in CMA in the meta-analysis conducted by Xiong et al. [61]. However, the authors did not confirm the usefulness of fecal calprotectin in the diagnosis and monitoring of cow’s milk allergy. Similarly, we did not observe significant differences in serum concentration of calprotectin between the groups of allergic and healthy children.

Although the results of this study, indeed, do not yet create a direct clinical tool allowing for immediate practical application, in our opinion, they constitute solid ground for further investigations in this promising area. Considering that diagnostics of food allergy, especially IgE-independent food allergy, is an important issue, the results of our study contribute to the search for markers facilitating diagnostics and monitoring effectiveness of therapy of this type of allergy. Having in mind the potential practical importance of the possible outcomes of subsequent research, further investigations seem to be necessary and fully justified.

This study has several limitations. The main limitation may be related to the cross-sectional nature of our study, small sample size, and short-term follow-up. However, the studied group is homogenous, all subjects were Caucasian children aged 3 to 6 years. All children were in the prepubertal period, which eliminated the puberty’s influence on the concentration of tested adipokines. Overall, the sample size of 64 is comparable to the scope of other published studies on CMA in children [9,41,42]. Despite the mentioned limitations, we can report significant alterations in pro-inflammatory adipokine levels in children with CMA and their significant correlations with CRP and WBC. To the best of our knowledge, we analyzed chemerin and NGAL concentrations in children with CMA for the first time. In this study, we assessed pro-inflammatory adipokine levels only once lacking the ability to demonstrate not only static but also their dynamic changes. We are planning to continue our study and include a group of newly diagnosed children before introducing a milk-free diet and to determine other adipokines, that have anti-inflammatory properties (adiponectin and omentin) in patients with cow’s milk allergy.

In conclusion, the use of a strict milk-free diet by children with CMA, resulting in the resolution of the disease’s clinical symptoms, does not seem to extinguish the inflammation induced by the allergy. The findings of elevated IL-6, TNF-α, resistin, chemerin and NGAL levels in patients with CMA, suggest that these cytokines seem to be involved in the generation of a low-grade proinflammatory environment observed in cow’s milk allergy. It is also possible that they could be used to monitor the effectiveness of the treatment. The obtained results will contribute to broadening the knowledge on the role of cytokines and adipokines and their interrelationships in the mechanisms of inflammation in children with allergies. However, further extensive investigations are required to explore the effect of cytokines on inflammation and its potential utility in clinical applications.

## Figures and Tables

**Table 1:** Baseline characteristics of the study participants.

	Children with Cow’s Milk Allergy (n = 64)	Healthy Children (n = 30)	*p*
Age (years) ^b^	4.3 (3.0–5.5)	5.0 (3.8–5.6)	0.164
Weight (kg) ^a^	16.7 ± 3.7	19.1 ± 3.9	0.016
Height (cm) ^a^	105.2 ± 11.4	111.2 ± 11.6	0.043
BMI (kg/m^2^) ^a^	14.9 ± 1.1	15.4 ± 1.7	0.281
BMI z-score ^a^	−0.74 ± 0.76	−0.62 ± 0.60	0.572

Data are expressed as ^a^ mean values and SD (standard deviation) or ^b^ median values and interquartile range (IQR); BMI—body mass index.

**Table 2:** Biochemical parameters associated with inflammation in children with CMA and healthy children.

Parameters	Children with CMA (n = 64)	Healthy Children (n = 30)	*p*
IL-6 (pg/mL) ^b^	0.92 (0.51–1.32)	0.71 (0.41–0.94)	0.049
IL-10 (pg/mL) ^b^	1.8 (1.0–2.4)	1.5 (1.0–1.8)	0.075
TNF-α (pg/mL) ^a^	13.2 ± 3.7	9.5 ± 2.0	0.000
Leptin (ng/mL) ^b^	0.62 (0.28–0.81)	0.81 (0.26–1.18)	0.086
Resistin (ng/mL) ^b^	17.2 (12.5–22.3)	14.3 (9.9–18.9)	0.037
Chemerin (ng/mL) ^b^	100.8 (85.3–112.0)	86.9 (80.4–97.7)	0.005
NGAL (ng/mL) ^b^	90.6 (75.6–119.5)	72.7 (64.7–77.6)	0.000
Calprotectin (ng/mL) ^b^	903.3 (617.0–1167.7)	1009.5 (770.1–1469.6)	0.229
CRP (mg/L) ^b^	0.47 (0.11–0.50)	0.41 (0.11–0.50)	0.899
WBC (10^9^/L) ^a^	7.62 ± 1.76	7.37 ± 1.91	0.572
Eosinophils (10^9^/L) ^a^	0.47 ± 0.30	0.20 ± 0.08	<0.001

Data are expressed as ^a^ mean values and standard deviation (SD) or ^b^ median values and interquartile range (IQR); IL-6—interleukin-6; IL-10—interleukin-10; TNF-α—tumor necrosis factor-α; NGAL—neutrophilic lipocalin associated with gelatinase; CRP—C-reactive protein; WBC—white blood cell count.

**Table 3:** Biochemical parameters associated with inflammation in groups of children IgE-mediated CMA and non-IgE-mediated CMA.

Parameters	IgE-Mediated CMA Patients (n = 41)	Non-IgE-Mediated CMA Patients (n = 23)	*p*
IL-6 (pg/mL) ^b^	0.83 (0.50–1.62)	0.70 (0.53–1.64)	0.021
IL-10 (pg/mL) ^b^	1.78 (0.97–2.44)	1.80 (1.28–2.41)	0.548
TNF-α (pg/mL) ^a^	13.18 ± 3.38	13.13 ± 4.35	0.968
Leptin (ng/mL) ^b^	0.48 (0.27–0.75)	0.40 (0.29–0.89)	0.545
Resistin (ng/mL) ^b^	17.04 (12.52–22.12)	17.46 (13.18–21.81)	0.837
Chemerin (ng/mL) ^b^	96.42 (84.34–114.22)	100.3 (87.49–109.88)	0.417
NGAL (ng/mL) ^b^	94.08 (79.45–119.04)	86.01 (74.32–118.42)	0.516
Calprotectin (ng/mL) ^b^	994.1 (684.9–1188.1)	774.9 (594.9–1000.7)	0.339
CRP (mg/L) ^b^	0.20 (0.12–0.46)	0.30 (0.16–0.83)	0.120
WBC (10^9^/L) ^a^	7.59 ± 1.78	7.67 ± 1.76	0.858
Eosinophils (10^9^/L) ^a^	0.57 ± 0.29	0.27 ± 0.14	<0.001

Data are expressed as ^a^ mean values and standard deviation (SD) or ^b^ median values and interquartile range (IQR); TNF-α—tumor necrosis factor α; NGAL—neutrophilic lipocalin associated with gelatinase; CRP—C-reactive protein; WBC—white blood cell count.

**Table 4:** Serum cytokine, calprotectin and adipokine concentrations in the subgroups of CMA patients with different clinical symptoms.

	Cutaneous (n = 7)	Respiratory (n = 30)	Gastrointestinal (n = 12)	Mixed (n = 15)
IL-6 (pg/mL)	0.94 (0.89–1.41)	0.99 (0.61–1.44)	0.83 (0.51–1.34)	0.87 (0.60–0.97)
IL-10 (pg/mL)	1.53 (0.68–2.58)	1.57 (1.01–1.91)	2.19 (1.19–2.54) *	1.97 (1.33–2.80)
TNF-α (pg/mL)	13.70 ± 4.09	12.99 ± 2.48	13.64 ± 4.13	13.74 ± 3.70
Calprotectin (ng/mL)	1250 (996–1480)	941 (690–1075) *	1140 (913–1293)	1001 (747–1507)
Chemerin (ng/mL)	95.1 (92.5–103.2)	96.4 (84.6–107.1)	100.3 (85.4–116.4)	107.1 (94.5–126.6)
Resistin (ng/mL)	14.2 (10.5–17.5)	16.6 (11.9–20.5)	13.1 (9.5–21.0)	18.9 (14.8–22.2)
NGAL (ng/mL)	74.8 (49.2–87.8)	91.5 (76.7–115.3)	87.1 (75.2–113.3)	93.5 (71.8–119.8) *
Leptin (ng/mL)	0.5 (0.4–0.7)	0.4 (0.3–0.7)	0.3 (0.2–0.6)	0.7 (0.4–0.8)

Data are expressed as mean values and standard deviation (SD) or median values and interquartile range (IQR); IL-6—interleukin-6; IL-10—interleukin-10; TNF-α—tumor necrosis factor-α; NGAL—neutrophilic lipocalin associated with gelatinase; * *p* < 0.05

**Table 5:** Serum concentration of biomarkers associated with inflammation in subgroups of children with CMA according to the duration of diet.

	Duration of Diet 6–12 Months (n = 11)	Duration of Diet 13–24 Months (n = 17)	Duration of Diet >24 Months (n = 36)
IL-6 (pg/mL)	0.56 (0.31–0.78)	0.83 (0.50–0.99)	1.01 (0.58–1.57)
IL-10 (pg/mL)	2.44 (1.97–3.03)	1.84 (0.89–2.36)	1.56 (1.04–1.97)
TNF-α (pg/mL)	13.3 ± 2.7	13.0 ± 3.6	13.4 ± 3.8
Calprotectin (ng/mL)	888 (648–1229)	788 (561–1055)	966 (707–1168)
Chemerin (ng/mL)	98.8 (88.0–116.9)	94.9 (86.7–107.0)	98.7 (86.3–113.1)
Resistin (ng/mL)	15.6 (14.2–23.7)	18.1 (11.8–23.3)	18.9 (12.5–25.7)
NGAL (ng/mL)	96.6 (79.9–136.5)	90.4 (75.7–119.3)	90.6 (80.4–108.8)
Leptin (ng/mL)	0.38 (0.32–0.51)	0.40 (0.26–1.02)	0.63 (0.29–0.94)

Data are expressed as mean values and standard deviation (SD) or median values and interquartile range (IQR); IL-6—interleukin-6; IL-10—interleukin-10; TNF-α—tumor necrosis factor-α; NGAL—neutrophilic lipocalin associated with gelatinase.

**Table 6:** Spearman correlations between serum cytokines and calprotectin concentrations and routinely determined parameters (CRP, WBC) in patients with cow’s milk allergy and in healthy children.

	IL-6		IL-10		TNF-α		Calprotectin	
	r	*p*	r	*p*	r	*p*	r	*p*
Children with cow’s milk allergy (n = 64):								
CRP	0.056	0.666	0.572	0.005	0.127	0.325	−0.117	0.388
WBC	−0.022	0.866	0.258	0.045	0.410	0.038	0.482	0.000
Healthy children (n = 30):								
CRP	0.215	0.199	0.188	0.403	0.085	0.912	−0.059	0.827
WBC	0.128	0.570	0.026	0.909	0.348	0.058	0.268	0.216

IL-6—interleukin 6; IL-10—interleukin 10; TNF-α—tumor necrosis α; CRP—C-reactive protein; WBC—white blood cell count.

**Table 7:** Spearman correlations between concentration of adipokines and routinely determined parameters (CRP, WBC) in patients with cow’s milk allergy and in healthy children.

	Leptin		Resistin		Chemerin		NGAL	
	r	*p*	r	*p*	r	*p*	r	*p*
Children with cow’s milk allergy (n = 64):								
CRP	0.209	0.108	0.030	0.818	0.434	0.000	−0.067	0.613
WBC	−0.180	0.168	0.060	0.641	0.145	0.262	0.388	0.002
Healthy children (n = 30):								
CRP	−0.109	0.658	0.546	0.009	0.639	0.001	0.137	0.543
WBC	−0.558	0.013	0.565	0.006	0.486	0.022	0.269	0.126

NGAL—neutrophilic lipocalin associated with gelatinase; CRP—C-reactive protein; WBC—white blood cell count.

## Data Availability

The data presented in this study are available upon reasonable request to the corresponding author.
